# Late onset of type 2 diabetes is associated with mitochondrial tRNA^Trp^ A5514G and tRNA^Ser(AGY)^ C12237T mutations

**DOI:** 10.1002/jcla.24102

**Published:** 2021-11-22

**Authors:** Liuchun Yang, Qinxian Guo, Jianhang Leng, Keyi Wang, Yu Ding

**Affiliations:** ^1^ Central Laboratory the Fourth School of Clinical Medicine Zhejiang Chinese Medical University Hangzhou China; ^2^ Central Laboratory the Affiliated Hangzhou First People's Hospital Zhejiang University School of Medicine Hangzhou China

**Keywords:** m.A5514G, m.C12237T, MIDD, mt‐tRNA mutations

## Abstract

**Background:**

Mitochondrial dysfunctions caused by mitochondrial DNA (mtDNA) pathogenic mutations play putative roles in type 2 diabetes mellitus (T2DM) progression. But the underlying mechanism remains poorly understood.

**Methods:**

A large Chinese family with maternally inherited diabetes and deafness (MIDD) underwent clinical, genetic, and molecular assessment. PCR and sequence analysis are carried out to detect mtDNA variants in affected family members, in addition, phylogenetic conservation analysis, haplogroup classification, and pathogenicity scoring system are performed. Moreover, the *GJB2*, *GJB3*, *GJB6*, and *TRMU* genes mutations are screened by PCR‐Sanger sequencing.

**Results:**

Six of 18 matrilineal subjects manifested different clinical phenotypes of diabetes. The average age at onset of diabetic patients is 52 years. Screening for the entire mitochondrial genomes suggests the co‐existence of two possibly pathogenic mutations: tRNA^Trp^ A5514G and tRNA^Ser(AGY)^ C12237T, which belongs to East Asia haplogroup G2a. By molecular level, m.A5514G mutation resides at acceptor stem of tRNA^Trp^ (position 3), which is critical for steady‐state level of tRNA^Trp^. Conversely, m.C12237T mutation occurs in the variable region of tRNA^Ser(AGY)^ (position 31), which creates a novel base‐pairing (11A‐31T). Thus, the mitochondrial dysfunctions caused by tRNA^Trp^ A5514G and tRNA^Ser(AGY)^ C12237T mutations, may be associated with T2DM in this pedigree. But we do not find any functional mutations in those nuclear genes.

**Conclusion:**

Our findings suggest that m.A5514G and m.C12337T mutations are associated with T2DM, screening for mt‐tRNA mutations is useful for molecular diagnosis and prevention of mitochondrial diabetes.

## Graphical abstract

In this study, we reported here the clinical, genetic and molecular features of a large Chinese pedigree with maternally inherited diabetes and deafness (MIDD). Through the application of PCR and direct sequence analysis, we identified two possible pathogenic mitochondrial tRNA mutations: tRNA^Trp^ A5514G and tRNA^Ser(AGY)^ C12237T, which were localized at extremely conserved nucleotides, altered secondary structure of tRNAs and may cause the failure in tRNAs metabolism, and subsequently lead to mitochondrial dysfunctions which were involved in the pathogenesis of MIDD.

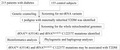

## 1 INTRODUCTION

Diabetes is a common endocrine disease in China, in particular, type 2 diabetes mellitus (T2DM) accounted for >10% of general population.^1^, ^2^ Although the pathophysiology of DM has not been fully elucidated, overwhelming evidence suggests that environmental, personal lifestyle, or nuclear genes mutations may influence T2DM pathogenesis.^3^, ^4^ Among these factors, some families are presented in maternally inherited pattern, indicating that mutations or variants in mitochondrial DNA (mtDNA) play critical roles in T2DM.^5^, ^6^

Human mitochondrial genome is a relative small molecule (16,569‐bp long) which encodes 13 polypeptides, 2 rRNAs (12S rRNA and 16S rRNA), and 22 tRNAs.^7^ Despite the fact that the entire mt‐tRNA genes account only for approximately 10% of total mitochondrial genome, more than 2/3 mitochondrial disease‐related pathogenic mutations are localized at tRNA genes.^8^, ^9^ Among these mutations, the A to G substitution at position 3243 appears to be the most common T2DM‐associated pathogenic mutation.^10^, ^11^, ^12^ Furthermore, several case‐control studies indicate that tRNA^Ile^ T4291C,^13^ tRNA^Glu^ A14692G, and T14709C mutations^14^, ^15^ are involved in the pathogenesis of T2DM.

Maternally inherited diabetes and deafness (MIDD) is a rare form of mitochondrial diabetes characterized by both DM and hearing loss. This disease can be resulted from genetic abnormalities in mtDNA, especially associated with tRNA^Leu(UUR)^ A3243G mutation.^16^ Moreover, MIDD typically affects metabolically active organs such as the endocrine pancreas and cochlea, and in some cases, also the retina, muscles, kidneys, and brain.^17^ However, the pathogenesis for the MIDD needs further elucidation.

To investigate the T2DM‐associated mtDNA mutations, our recently screened the mtDNA mutations in a cohort of 215 diabetic patients and 155 controls. Consequently, a four‐generation family with MIDD is identified in this case‐control study, to explore the contributions of mitochondrial dysfunction to DM, we perform PCR‐Sanger sequencing to analyze the mutations in whole mitochondrial genome.

Moreover, more than 160 loci, around 119 genes have been identified in patients with non‐syndromic hearing loss.^18^ In particular, Gap junctions (*GJs*) are intercellular channels that allow small molecules of the cytoplasm of a cell to be directed to the adjacent cell, including ions such as K^+^, Na^+^, and Ca2^+^. Connexins *GJB* contains 21 isoforms in humans, including *GJB2* (Cx26), *GJB3* (Cx31), and *GJB6* (Cx30).^19^ It has been suggested that several mutations such as c.235delC in *GJB2*, A194T in *GJB3*, 150‐kb large deletion in *GJB6* are the important causes for non‐syndromic hearing loss in many populations worldwide.^20^, ^21^, ^22^ In addition, *TRMU* is a nuclear gene crucial for mtDNA translation by encoding tRNA 5‐methylaminomethyl‐2‐thiouridylate methyltransferase, which thiolates mt‐tRNA.^23^Previous study suggested that mutation in *TRMU* may modulate the clinical expression of deafness‐associated mitochondrial 12S rRNA mutations.^24^ To see whether *GJB2*, *GJB3*, *GJB6*, and *TRMU* contributed to genetic susceptibility to deafness, we analyze the mutations in these nuclear genes by direct sequencing.

## 2 MATERIALS AND METHODS

### 2.1 Pedigree information and clinical assessments

**FIGURE 1:**
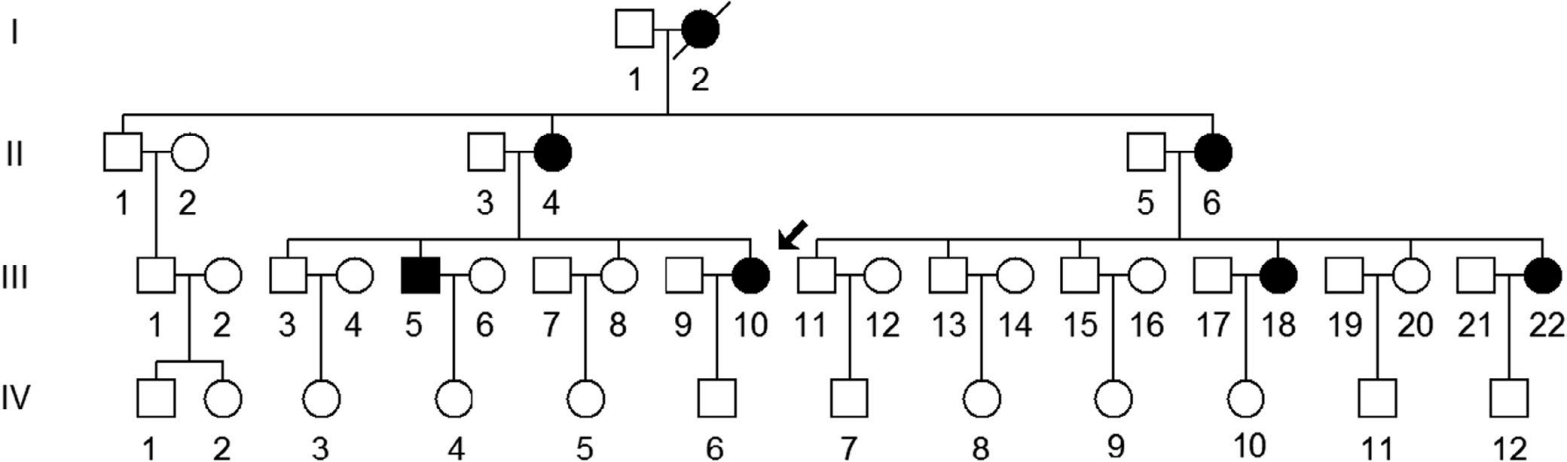
One Han Chinese family with T2DM, arrow indicated the proband, the affected diabetes patients were marked as filled symbols

From January 2019 to January 2021, we enrolled 215 subjects with diabetes and 155 controls from Hangzhou First People's Hospital, as shown in Figure 1, a large Chinese pedigree with T2DM was ascertained during this mutational screening program. We first invited the members of this family to participate for this study, the blood samples, family history, and detailed personal information were collected. This study was approved by the Ethics Committee of Hangzhou First People's Hospital (Approval Number: 2020‐004‐01), and each participant provided their written informed consent. Moreover, 155 healthy subjects including 70 males and 85 females, aged from 38 to 50 years were recruited from the Healthy Examination Center of our hospital as controls. These controls were healthy subjects without any diseases; whereas the subjects had a family history of mitochondrial diseases will be excluded.

The diagnosis criteria of DM were based on the standard proposed by the American Diabetes Association^25^: (1) The fast plasma glucose (FPG) level ≥7.0 mmol/L; (2) The 2‐h plasma glucose level ≥11.1 mmol/L after the oral glucose tolerance test (OGTT); (3) The concentration of hemoglobin A1c (HbA1c) >6.5%.

Body mass index (BMI) was calculated by as the body weight (kg) divided by the square of the height (m^2^). Obesity was defined using the BMI for Chinese adults: normal: 18.5–24 (kg/m^2^), overweight: 24–28 (kg/m^2^), and obese ≥28 (kg/m^2^). Moreover, we monitored the blood pressure (BP) by using an electronic sphygmomanometer, according to the protocol as previously described.^26^ The systolic BP ≥140 mmHg or the diastolic BP ≥90 mmHg was regarded as hypertension.^27^ For biochemical assessment, serum FPG was determined by the regular laboratory methods (Beckman Coulter AU5800). In addition, the OGTT was carried out by measurement of plasma glucose concentrations at 0 and 2‐h after 75‐g glucose administration, while plasma insulin (0 h) and C‐peptide (0 h) were measured by chemiluminescent immunometric assay (IMMULITE^®^, Siemens).^28^ Moreover, the audiological examination was assessed to evaluate the hearing function, which was calculated on the basis of results of pure‐tone audiometry (PTA). The degrees of hearing loss were divided into 5 groups: (1) PTA<26 Decibel (dB): normal hearing; (2) 26 dB<PTA<40 dB: mild; (3) 41 dB<PTA<70 dB: moderate; (4) 71 dB<PTA<90 dB: severe; and (5): PTA>90 dB: profound.^29^ In addition, visual acuity (VA) was evaluated to see the degree of vision loss, which was as follows: (1) VA>0.3: normal; (2) 0.1<VA<0.3: mild; (3) 0.05<VA<0.1: moderate; (4) 0.02<VA<0.05: severe; and (5) VA<0.02: profound.^30^

### 2.2 Screening for mtDNA mutations or variants

To detect the mtDNA variants, genomic DNA was extracted from blood of each participant by using Paxgene Blood DNA Isolation kits (QIAGEN). Briefly, 24 primers were used to amplify whole mitochondrial genomes from affected subjects (II‐4, II‐6, III‐5, III‐10, III‐18, and III‐22), according to the protocol as described previously.^31^ Furthermore, the ABI 3700 DNA instrument was employed to analyze the sequences by comparing with the revised Cambridge sequences (rCRS, GenBank accession number: NC_012920.1).^32^ The DNA STAR software package version 5.01 (Madison) was used to detect mtDNA mutations or variants.

### 2.3 Evolutionary conservation assessment

To analysis the potential pathogenicity of a candidate mtDNA mutation, phylogenetic conservation assessment was carried out. We chose 12 vertebrate species and then compared with human mtDNA variant at a certain position to see the degree of conservation index (CI).^33^ The CI ≥75% was regarded to be functional potential.^34^ Furthermore, mitochondrial haplogroup was classified according to the study by Kong et al.^35^

### 2.4 Bioinformatics analysis

With the purpose of understanding the molecular pathogenesis of m.A5514G and m.C12237T mutations, the online RNA Fold Webserver was used to determine the structural alternation of tRNA^Trp^ and tRNA^Ser(AGY)^ with and without these mutations (http://rna.tbi.univie.ac.at/cgi‐bin/RNAfold.cgi). ^36^ The wild‐type sequence for tRNA^Trp^ gene was 5'‐AGAAATTTAGGTTAAATACAGACCAAGAGCCTTCAAAGCCCTCAGTAAGTTGCAATACTTAATTTCTG‐3’; while the sequence of tRNA^Trp^ with m.A5514G mutation was 5'‐AGGAATTTAGGTTAAATACAGACCAAGAGCCTTCAAAGC CCTCAGTAAGTTGCAATACTTA ATTTCTG‐3'. Moreover, the wild‐type version of tRNA^Ser(AGY)^ was 5'‐GAGAAAGCTCACAAGAACTGCTAACTCATGCCC CCATGTCTAACAACATG GCTTTCTCA‐3’, and the sequence of tRNA^Ser(AGY)^ with m.C12237T mutation was 5'‐GAGAAAGCTCACAAGAACTGCTAAC TCATGTCCCCATGTCTAACAACAT GGCTT TCTCA‐3'. In addition to the secondary structure alternation, RNA Fold also provided the minimum free energy (MFE) of each tRNA.^37^

### 2.5 The pathogenicity scoring system

To identify the potential pathogenic mt‐tRNA mutations, we used the following criteria: (1) presented in <1% of the healthy controls; (2) CI ≥75%, as proposed by Ruiz‐Pesini and Wallace^34^; (3) potential to cause structural and functional alterations; and (4) a score of ≥7 points under an established pathogenicity scoring system.^38^ If the total scores of a mt‐tRNA mutation were less than 6, it belonged to “neutral polymorphism,” if the scores were 7–10, it was “possible pathogenic,” whereas the scores were more than 11, it was classified as “definitely pathogenic.”

### 2.6 Analysis of *GJB2*, *GJB3*, *GJB6*, and *TRMU* genes mutations

To see the contributions of nuclear genes to deafness expression, we conducted a mutational screening for *GJB2*, *GJB3*, *GJB6*, and *TRMU* genes in 6 affected patients from this pedigree (II‐4, II‐6, III‐5, III‐10, III‐18, and III‐22). The primers for amplifying *GJB2* gene were forward‐5'‐TATGACACTCCCCAGCACAG‐3’ and reverse‐5'‐GGGCAATGCTTAAACTGGC‐3’; the primers for amplification of *GJB3* gene were forward‐5'‐GTCACCTATTCATTCATACGATGG‐3’ and reverse‐5'‐TCACTCAGCCCCTGTAGGAC‐3’; the primers sequences for amplifying *GJB6* were forward‐5'‐CCTTAAAATAAAGTTGGCTTCAG‐3’, reverse‐5'‐GGAACTTTCAGGTTGGTATTG‐3’; the primers for *TRMU* exon 1 were forward‐5'‐ACAGCGCAGAAGAAGAGCAGT‐3’ and reverse‐5'‐ACAACGCCACGACGGACG‐3'. PCR products were purified and subsequently sequenced by ABI3700 DNA instrument, the sequence data were compared with the wild‐type versions of *GJB2*, *GJB3*, *GJB6*, and *TRMU* sequences (GenBank accessible numbers: M86849, AF052692, NG_008323, and AF_448221, respectively) to detect the mutations or variants.^39^, ^40^, ^41^, ^42^

## 3 RESULTS

### 3.1 Clinical findings

We ascertained a Chinese family with MIDD from Hangzhou First People's Hospital (Figure 1). Detailed information was obtained from each subject of this pedigree, as well as the family history of diabetes or deafness. After the genetic counseling, we noticed that the proband (III‐10) was a 45 years old woman, who suffered from T2DM one year ago before the administration of our hospital. Laboratory examination showed that she was a diabetes carrier. Of 18 matrilineal relatives, 6 (1 male and 5 female) individuals suffered from diabetes. The onset of T2DM ranged from 40 to 70 years (mean age: 52). Furthermore, 3 individuals (II‐4, II‐6, and III‐5) developed both diabetes and hearing loss, in particular, the subject (II‐4) was a hypertension, deafness, and vision impairment carrier. Notably, subjects (II‐4 and II‐6) exhibited hearing loss most probably due to their great age, because during aging, mitochondrial ROS increased and impaired the mitochondrial function.^43^ However, these subjects did not have other clinical disorders, including coronary heart disease, cancer, or infectious diseases (Table 1).

**TABLE 1:** Summary of clinical and biochemical data of members in this family with T2DM

Subjects	Gender	BMI (kg/m^2^)	Age at onset (yrs)	Age at test (yrs)	Glucose (0h, mmol/L)	Glucose (2‐h, mmol/dL)	HbA1c (%)	Fast insulin (uU/mL)	C‐Peptide (nmol/L)	BP (mmHg)	PTA (Left/right ear, dB)	Level of hearing loss	Visual acuity (Left/right eye)	Level of vision loss	Clinical presentations
II‐4	Female	23.2	70	75	16.3	1.78	7.6	14.66	2.30	150/95	55/75	Severe	0.2/0.1	Mild	Diabetes, hypertension, deafness, vision loss
II‐6	Female	22.5	68	78	15.0	1.72	7.4	12.22	0.76	140/90	90/95	Profound	0.1/0.1	Moderate	Diabetes, hypertension, deafness, vision loss
III‐5	Male	27.5	50	50	8.8	1.66	7.0	4.70	1.23	130/80	55/30	Mild	0.5/0.4	Normal	Diabetes, deafness
III‐10	Female	28.0	44	45	7.1	1.33	6.8	6.91	0.98	110/85	20/20	Normal	0.3/0.4	Normal	Diabetes
III‐18	Female	26.5	42	48	8.0	1.54	7.0	10.3	0.82	125/80	20/18	Normal	0.4/0.4	Normal	Diabetes
III‐22	Female	24.0	40	44	7.5	1.22	6.9	15.1	1.08	130/85	15/20	Normal	0.5/0.5	Normal	Diabetes
III‐21	Male	21.5	/	48	5.1	0.73	5.3	3.9	1.2	130/80	15/15	Normal	0.4/0.5	Normal	Normal

Abbreviations

BMI: body mass index; HbA1c: glycosylated hemoglobin; BP: blood pressure; PTA: pure‐tone audiometry; dB: decibel

### 3.2 Screening for mtDNA mutations

**FIGURE 2:**
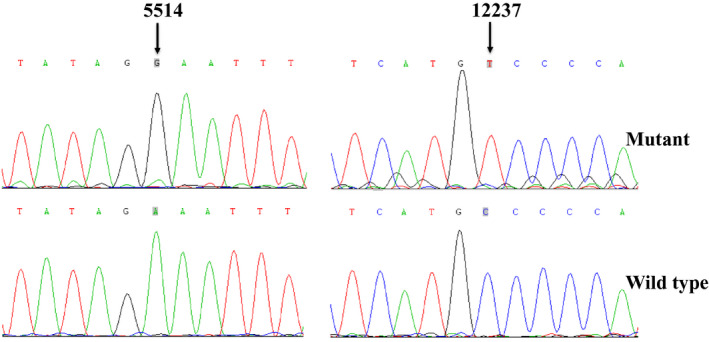
Identification of tRNA^Trp^ A5514G and tRNA^Ser(AGY)^ C12237T mutations by Sanger sequencing

**FIGURE 3:**
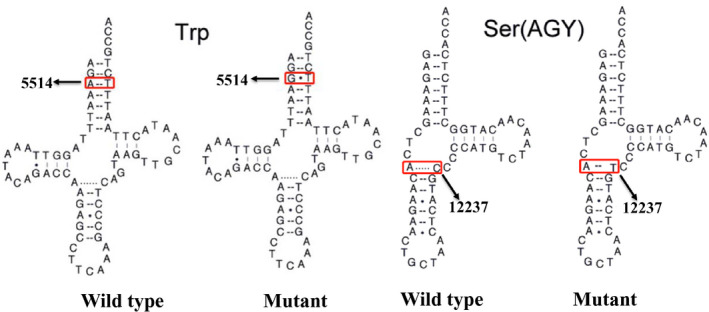
Cloverleaf structures of tRNA^Trp^ and tRNA^Ser(AGY)^. Arrows indicate the positions of m.A5514G and m.C12237T mutations in mt‐tRNA genes

**FIGURE 4:**
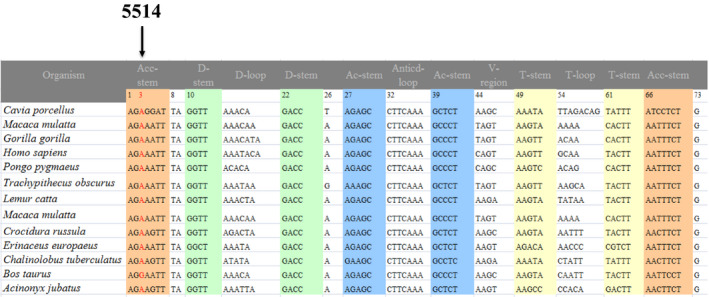
Sequence alignment of tRNA^Trp^ gene from different species, arrow indicates the position 3, corresponding to the m.A5514G mutation

Owing to the maternally inheritance, we screened the mtDNA mutations in 6 affected matrilineal relatives of this family. PCR‐Sanger sequence revealed 28 mtDNA variants which belonged to East Asia haplogroup G2a.^35^ Among these, 8 variants were identified in D‐loop, 2 variants were found in 12S rRNA (m.A750G and m.A1438G), 1 variant occurred at 16S rRNA (m.A2708G), 2 potential pathogenic mutations were found in tRNA genes (m.A5514G and m.C12237T) (Table 2). Whereas others were resided at oxidative phosphorylation (OXPHOS)‐encoding genes. In addition, 6 missense mutations were identified: m.G8584A (Ala to Thr) and m.A8860G (Thr to Ala) in *A6* gene, m.A10398G (Thr to Ala) in *ND3* gene, m.G13928C (Ser to Thr) in *ND5* gene, m.C14766T (Thr to Ile) and m.A15326G (Thr to Ala) in *CytB* gene. To further evaluate their pathogenicity, evolutionary conservation of each variant was assessed including mouse,^44^ bovine,^45^ and *Xenopus laevis*.^46^ We found that except for tRNA^Trp^ A5514G, tRNA^Ser(AGY)^ C12237T mutations (Figures 2, 3, 4), other mtDNA variants may not be pathogenic since they either occurred in control group or had very low degrees of CIs.

**TABLE 2:** mtDNA sequence alternation in this pedigree with diabetes

Gene	Position	Replacement	rCRS	Conservation (H/B/M/X)^a^	CI (%)	Previously reported^b^
D‐loop	73	A to G	A			Yes
	204	T to C	T			Yes
	215	A to G	A			Yes
	263	A to G	A			Yes
	310	T to CTC	T			Yes
	16182	A to C	A			Yes
	16189	T to C	T			Yes
	16519	T to C	T			Yes
12S rRNA	750	A to G	A		97.78	Yes
	1438	A to G	A	A/A/A/G	86.67	Yes
16S rRNA	2706	A to G	A	A/G/A/A	84.44	Yes
ND1	3970	C to T	C		80.0	Yes
ND2	4769	A to G	A		24.44	Yes
	5441	A to G	A		31.1	Yes
tRNA^Trp^	5514	A to G	A	A/A/A/A	100	Yes
CO1	6962	G to A	G		100	Yes
	7028	C to T	C		100	Yes
A6	8584	G to A (Ala to Thr)	G	A/V/V/I	17.8	Yes
	8860	A to G (Thr to Ala)	A	T/A/A/T	71.1	Yes
ND3	10398	A to G (Thr to Ala)	A	T/T/T/A	51.1	Yes
ND4	11719	G to A	G		97.8	Yes
tRNA^Ser(AGY)^	12237	C to T	C	C/C/C/C	100	Yes
ND5	12822	C to T	C		22.2	Yes
	13928	G to C (Ser to Thr)	G	S/T/S/T	11.1	Yes
CytB	14766	C to T (Thr to Ile)	C	T/S/T/S	48.9	Yes
	15301	G to A	G		95.5	Yes
	15326	A to G (Thr to Ala)	A	T/M/I/I	17.8	Yes
	15784	T to C	T		100	Yes

Abbreviation: CI, conservation index.

^a^ Conservation of amino acid for polypeptides or nucleotide for rRNAs, in human (H), mouse (M), bovine (B), and *Xenopus laevis* (X).

^b^ See http://www.mitomap.org andhttp://www.genpat.uu.se/mtDB/

As shown in Figure 3 and Table 3, the A‐to‐G substitution at position 5514 was localized at acceptor arm of tRNA^Trp^ gene, disrupting a very conserved Watson‐Crick base‐pairing (3A‐70T). Conversely, m.C12337T mutation was believed to create a new Watson‐Crick base‐pairing (11A‐31T). Thus, it was anticipated that the m.A5514G and m.C12237T mutations may cause the structural alternation of the corresponding tRNAs and affect their functions.

**TABLE 3:** Molecular characterizations of tRNA^Trp^ A5514G and tRNA^Ser(AGY)^ C12237T mutations identified in this family

tRNA species	Nucleotide changes	Number of nucleotides in tRNA	Location in tRNA	Watson‐Crick base‐pairing^a^	CI (%)	MFE (wild type) kcal/mol	MFE (Mutant) kcal/mol	Disease association
tRNA^Trp^	A5514G	3	Acceptor arm	3A−70T↓	92.3	−10.82	−10.88	Deafness; Multiple mitochondrial respiratory chain enzyme defects
tRNA^Ser(AGY)^	C12237T	31	Variable region	11A−31T↑	84.6	−15.55	−14.96	Creutzfeldt‐Jakob disease

Abbreviations: CI, conservation index; MFE, minimum free energy.

^a^ Classic Watson‐Crick base pairing: created (↑) or abolished (↓).

### 3.3 m.A5514G and m.C12237T mutations affected tRNAs secondary structure

**FIGURE 5:**
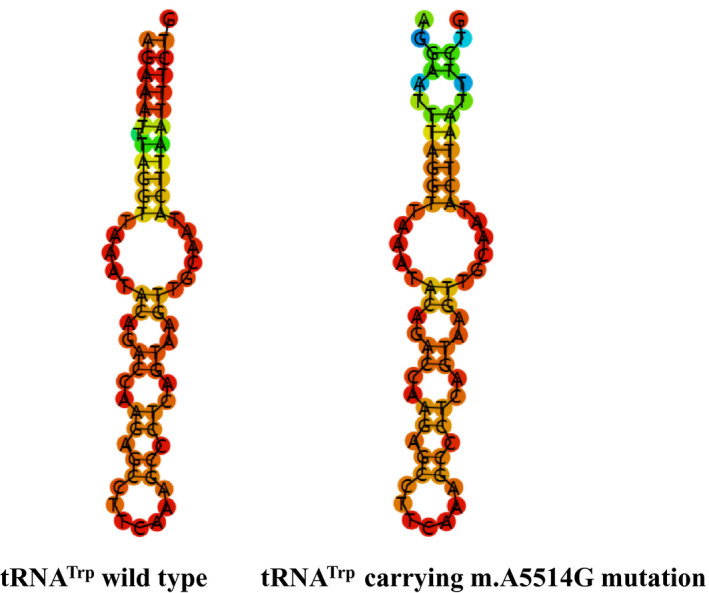
Bioinformatics analysis of tRNA^Trp^ structure with and without m.A5514G mutation

**FIGURE 6:**
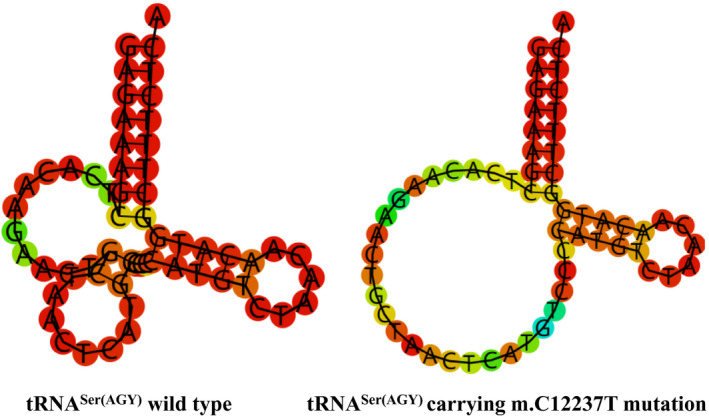
Bioinformatics analysis of tRNA^Ser(AGY)^ structure with and without m.C12237T mutation

To see the effects of m.A5514G and m.C12237T mutations on tRNAs structure, we used RNA Fold program to analyze the secondary structure of tRNA^Trp^ and tRNA^Ser(AGY)^ with and without these mutations.^36^ As shown in Figures 5 and 6, we noticed that m.A5514G and m.C12237T mutations influenced the structures of tRNA^Trp^ and tRNA^Ser(AGY)^, respectively, suggesting that they may have functional potential.

### 3.4 m.A5514G and m.C12237T mutations were “possibly pathogenic” associated with MIDD

As shown in Table 4, based on the classic pathogenicity scoring system,^38^ the total scores of m.A5514G and m.C12237T mutations were both 8 points, which belonged to “possibly pathogenic” mutations for MIDD.

**TABLE 4:** Predicted pathogenicity of mt‐tRNA^Trp^ A5514G and tRNA^Ser(AGY)^ C12237T mutations

Scoring criteria	m.A5514G mutation	Score/20	m.C12237T mutation	Score/20	Classification
More than one independent report	Yes	2	Yes	2	
Evolutionary conservation of the base pair	No changes	2	No changes	2	
Variant heteroplasmy	No	0	No	0	≤6 points: neutral polymorphisms
Segregation of the mutation with disease	Yes	2	Yes	2	
Histochemical evidence of mitochondrial disease	No evidence	0	No evidence	0	7~10 points: possibly pathogenic
Biochemical defect in complex I, III, or IV	No	0	No	0	11–13 points (not including evidence from single fiber, steady‐state level, or trans‐mitochondrial cybrid studies): probably pathogenic
Evidence of mutation segregation with biochemical defect from single‐fiber studies	No	0	No	0	
Mutant mt‐tRNA steady‐state level or evidence of pathogenicity in trans‐mitochondrial cybrid studies	Weak evidence	2	Weak evidence	2	≥11 points (including evidence from single fiber, steady‐state level, or trans‐mitochondrial cybrid studies): definitely pathogenic
Maximum score	Possibly pathogenic	8	Possibly pathogenic	8	

### 3.5 Mutational analysis of nuclear genes

To see whether nuclear genes (*GJB2*, *GJB3*, *GJB6*, and *TRMU*) mutations played active roles in clinical expression of hearing impairment, we initiated a mutational analysis of the exons of *GJB2*, *GJB3*, *GJB6*, and *TRMU* in matrilineal relatives of this pedigree. However, we did not find any functional variants in these genes.

## 4 DISCUSSION

Mutations in mtDNA were the important causes for MIDD, currently, the clinical features of MIDD were often variable due to the heteroplasmy and subsequent segregation of the mutated mtDNA.^47^ Diagnosis and prediction of MIDD prognosis were difficult for providers based on phenotypic features alone because of the large variation of heteroplasmic mtDNA inheritance.^48^ At the cellular level, the β‐cell required large amounts of ATP to produce insulin. The impaired mitochondrial functions caused by mtDNA mutations decreased ATP production and increased ROS level leading to abnormal β‐cell functions, loss of β‐cell mass, and eventually insulin deficiency.^49^

To see the relationship between mt‐tRNA mutations and MIDD, the current study described a large maternally inherited MIDD pedigree which harbored both tRNA^Trp^ A5514G and tRNA^Ser(AGY)^ C12237T mutations. In fact, among 18 matrilineal relatives, six of them developed T2DM at different age at onset. In particular, the onset of T2DM varied from 40 to 70 years (mean age: 52). Despite that these subjects also developed hearing loss and vision impairment, the pattern of transmission was consistent with maternally inheritance. Interestingly, family members from the second generation (II‐4 and II‐6) to third generation (III‐5, III‐10, III‐18, and III‐22) manifested earlier onset of DM, indicating that mitochondrial dysfunction may be the molecular basis for this disease.

Mutational screening for the complete mtDNA genes of the affected individuals led us to identify 28 polymorphisms which belonged to human mitochondrial haplogroup G2a.^35^ Of these, m.A5514G mutation may have functional impact on tRNA metabolism on the following lines of evidence: first, this mutation resided at the acceptor arm of tRNA^Trp^ (position 3), which had a high level of CI.^34^ Secondly, m.A5514G mutation resulted the A3G transition of tRNA^Trp^, causing the misreading and recognition by RNase P.^50^ Interestingly, m.T7512C mutation which occurred at the same position of tRNA^Ser(UCN)^ was implicated to be associated with MELAS.^51^, ^52^ Importantly, m.T7512C mutation also caused a strong reduction of tRNA^Ser(UCN)^ steady‐state level and affected the post‐transcriptional modification of this tRNA.^53^ Therefore, m.A5514G mutation was similar to m.T7512C mutation, may also influence the tRNA^Trp^ metabolism and lead to mitochondrial dysfunction.

Moreover, m.C12237T mutation occurred at position 31 in the variable region of tRNA^Ser(AGY)^ and was expected to form a novel Watson‐Crick base‐pairing (11A‐31T). Interestingly, m.A12308G mutation which was located at the position 31 in tRNA^Leu(CUN)^, had been found to be associated with cardiomyopathy,^54^ metabolic syndrome,^55^ and increasing the risk of stroke.^56^ Therefore, we believed that the m.C12237T mutation, which was similar to m.A12308G mutation, most probably led to the failure in tRNAs metabolism via affecting its secondary structure, and subsequently impair the mitochondrial functions.^57^

Based on these observations, we proposed that the possible molecular mechanisms underlying m.A5514G and m.C12237T mutations in the phenotypic expression of MIDD may be as follows: first, the mutations disrupted the secondary structures of tRNAs and subsequently resulted the failure in tRNAs metabolism, such as reducing tRNA steady‐state level, aminoacylation ability, affecting 3’ end processing, or its chemical modifications.^58^ These biochemical processes will lead to the impairment of mitochondrial protein translation and influence the respiratory chain functions. As a result, these mutations led to mitochondrial dysfunctions which caused the pancreatic β‐cell apoptosis or necrosis,^59^, ^60^ and involved in the pathogenesis of MIDD in this pedigree. At the same time, the absent of functional variants in *GJB2*, *GJB3*, *GJB6*, and *TRMU* genes suggested that these genes may not play putative roles in the phenotypic manifestation of MIDD, therefore, the combination of tRNA^Trp^ A5514G and tRNA^Ser(AGY)^ C12237T mutations may be responsible for MIDD in this pedigree. The main limitation of this study was the relatively small sample sizes, further studies including more patients with DM were needed to verify the conclusions.

## CONFLICT OF INTEREST

None.

## ETHICS APPROVAL

The Ethics Committee of Hangzhou First People's Hospital approved this study (No. 2020‐004‐01).

## Data Availability

All data generated or analyzed during this study are included in this article. Further enquiries can be directed to the corresponding author.
